# Differential cell counts using center-point networks achieves human-level accuracy and efficiency over segmentation

**DOI:** 10.1038/s41598-021-96067-3

**Published:** 2021-08-19

**Authors:** Sarada M. W. Lee, Andrew Shaw, Jodie L. Simpson, David Uminsky, Luke W. Garratt

**Affiliations:** 1 Perth Machine Learning Group, Perth, WA 6000 Australia; 2 School of Medicine and Public Health, University of Newcastle, Callaghan, NSW 2308 Australia; 3 Data Institute, University of San Francisco, San Francisco, CA 94117 USA; 4 Priority Research Centre for Healthy Lungs, University of Newcastle, Callaghan, NSW 2308 Australia; 5 Department of Computer Science, University of Chicago, Chicago, IL 60637 USA; 6 Wal-yan Respiratory Research Centre, Telethon Kids Institute, University of Western Australia, Nedlands, WA 6009 Australia

**Keywords:** Biomarkers, Pathology

## Abstract

Differential cell counts is a challenging task when applying computer vision algorithms to pathology. Existing approaches to train cell recognition require high availability of multi-class segmentation and/or bounding box annotations and suffer in performance when objects are tightly clustered. We present differential count network (“DCNet”), an annotation efficient modality that utilises keypoint detection to locate in brightfield images the centre points of cells (not nuclei) and their cell class. The single centre point annotation for DCNet lowered burden for experts to generate ground truth data by 77.1% compared to bounding box labeling. Yet centre point annotation still enabled high accuracy when training DCNet on a multi-class algorithm on whole cell features, matching human experts in all 5 object classes in average precision and outperforming humans in consistency. The efficacy and efficiency of the DCNet end-to-end system represents a significant progress toward an open source, fully computationally approach to differential cell count based diagnosis that can be adapted to any pathology need.

## Introduction

Cytopathology is a common technique to visualise cells through a glass slide preparation, created by cytocentrifugation of a cell suspension followed by treatment with histological stains. Used for decades to visualise cells in a variety of clinical samples, including blood, bone marrow, sputum and cerebrospinal fluid^1^, this technique has advantages of very low cost, minimal reagent requirements, visual representation of the cell and rapid throughput. Yet the analysis of prepared cytocentrifugation slides remains surprisingly reliant upon labor-intensive assessment by trained humans. This presents a number of methodological variations including human error, the area observed and the number of cells counted^2,3^. Although the rise of deep learning has produced remarkable computer vision results in both the medical imaging space and cell nuclei identification^4–7^, commercial development of these techniques for clinical cytopathology is limited. The CellaVision${}^\circledR$ platform that was developed to assess hematological malignancies through computer vision has been available for over a decade^8^. However, the infrastructure and cost requirements of this complete hardware solution can present obstacles, particularly for applications outside specialised hospital settings.

An alternative is to embrace imaging hardware already existing in many laboratories and provide a software platform for researchers and healthcare professionals alike to train analytical solutions for their cytopathology needs, using their cytopathology preparations. Cell orientated datasets published to date have been based upon histological or cell culture images^9–15^ which typically feature homogenous cell populations and often not suited to specialised training tasks. Further considerations that might drive the need for specialised dataset generation include unique preparation practices, so cytopathology images may have significant variance in color, size/resolution and cell content specific to the laboratory^1^, or institutions may be ethically restricted from making data available externally. Yet the most critical bottleneck in generating cytopathology datasets is the key process of image annotation, especially when multiple object classes are present and labelling for ground truth classification can only be performed by trained experts. Keypoint detection is a recent annotation approach that promises much faster generation of ground truth image data. Instead of estimating 4-point bounding boxes for each object, these keypoint detection frameworks predict various keypoints along the object. These keypoints can be the extreme outer points as with ExtremeNet^16^, or they can be the centre points and the respective corners as with CornerNet^17^. Keypoint detection (CenterNet) has shown to be a simpler and yet often more accurate approach than bounding box or segmentation, even in complex image tasks such as real-time inference from video streaming^18^.

In respiratory medicine, differential immune cell count is one such multiple classification problem ideal for computer vision. While some blood based image datasets are publicly available^19,20^, morphological features of immune cell subsets can vary following their migration into the lung and activation by the airway environment. In chronic diseases, excess accumulation and cell death may further alter morphological characteristics or present new image artifacts such as mucous or cell debris. There are currently no well annotated digital datasets from respiratory cytopathology. This has prevented a true understanding of variability by human assessors, which may be masked by the inability of microscope based assessments to directly compare the same area. More significantly, digitised images are needed for the development of automated differential immune cell count pipelines that are specialised to respiratory samples. The CellaVision${}^\circledR$ platform was recently tested for assessing asthma cytopathology using the “body fluid” reference database, where it demonstrated reasonable capacity to match categorical inflammation assessments by trained human pathologists^21^, highlighting the excellent potential for deep learning based image analysis to facilitate clinical care. To realise this potential, we sought to empower pathology and medical experts to create highly-annotated cell image datasets that meet their needs at minimal labor expense and agnostic to the imaging platform. We investigated whether keypoint annotation, which has been used to identify cell nuclei in existing annotated datasets^10,11^, could successfully train a deep learning based immune cell classification tool, using respiratory samples as demonstration case. The result was Differential Count Network (“DCNet”). A new centre point classifier that enables maximally efficient annotation of multiple class labels for a rapid end-to-end deep learning system, which uses *whole cell features rather than just nuclei*. To this end, we make the following contributions:

1. We built a well annotated multi-class cytospin dataset and highlight the trade-offs of different annotation methods.
2. We established the human performance baseline through interperson variability testing on our ground truth cytospin dataset.
3. The creation of DCNet, which matches or surpasses human performance on detection of each immune cell class in our cytospin dataset.
4. Benchmark DCNet against other object detection models on a public cell dataset and show improvements in both efficiency and accuracy.

## Results

### Cytospin dataset and efficient annotation methods

**Figure 1:**
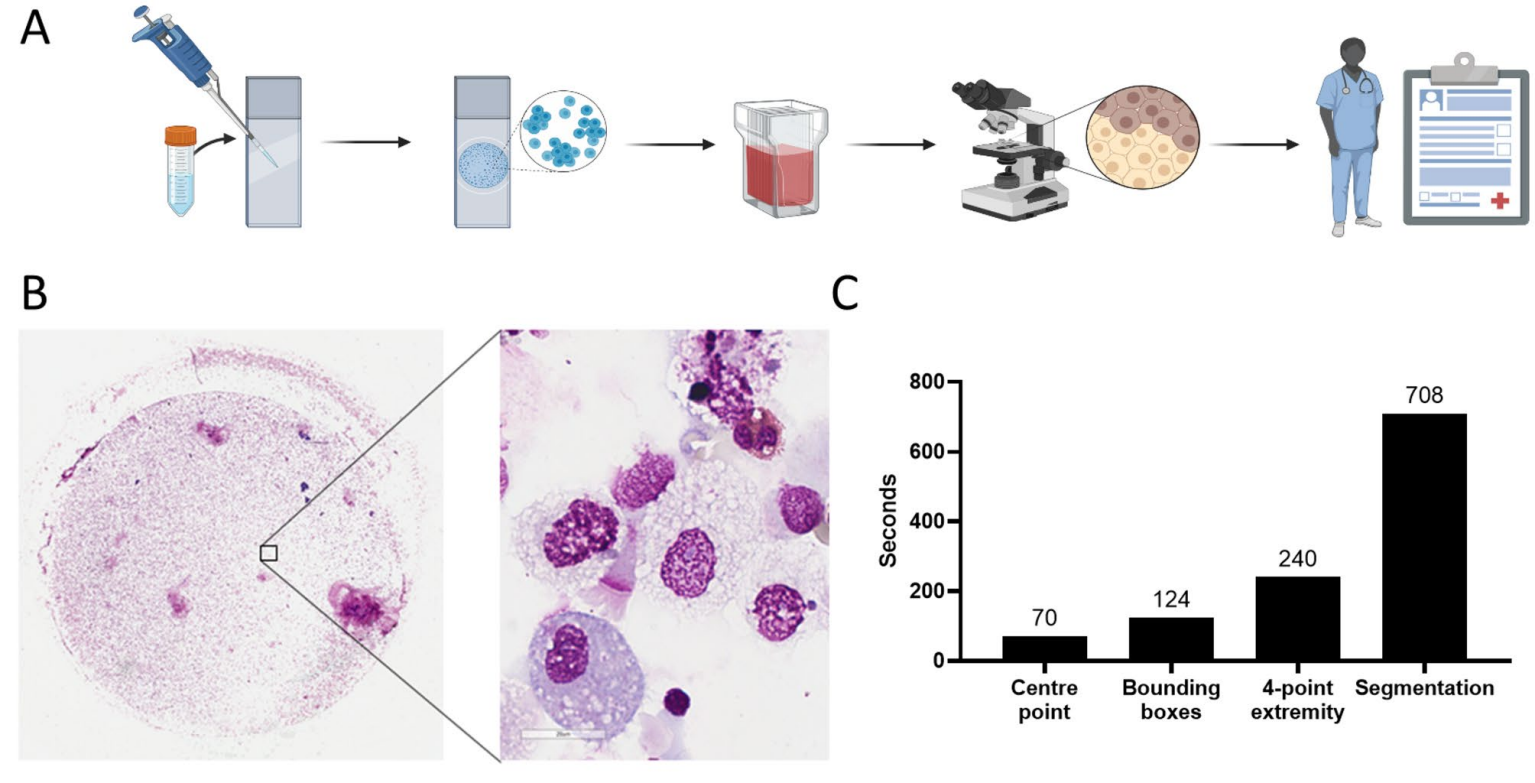
Cytospin generation and annotation. (**A**) The cell fraction of bronchoalveolar lavage or sputum was applied to glass slides by centrifugation, stained with May Grumwald Giemsa based solutions and then digitally imaged by microscopy. (**B**) Representative digital images at × 4 (overview) and × 100 magnification (inset). (**C**) Average time in seconds for assessors to annotate a $\times$ 100 magnification sample image tiles by four separate annotation modalities. Lower is better. Figure 1A created with BioRender.com.

A total of 19 cytospins were used in this study (Table 1) that were generated according to standard centrifugation practice (Fig. 1A). To develop a relevant real-world dataset for the assessment of annotation burden and performance of the DCNet classifier, samples were selected to specifically reflect a range of clinical respiratory samples, in terms of density and diversity of the cell populations, non-object debris and intensity of histological staining.

**Table 1:** Source of clinical respiratory samples and variations in cytospin preparation.

	Cohort 1	Cohort 2
Age group	Children $<7$ years	Adults 18–65 years
Respiratory disease	Cystic fibrosis	Asthma
Specimen collection	Bronchoalveolar lavage	Induced sputum
Estimated cell content	3–6 $\times\, 10^{5}$ Total cells	3–6 $\times \,10^{5}$ total cells
Stain applied	Kwik-Diff™	May–Grumwald Giemsa
Total cytospins	7	12
Total image tiles for annotation	280	480

At × 100 magnification, each cytospin generated approximately $1 \times 10^{4}$ image tiles sized $1024 \times 1024$ pixels (example shown in Fig. 1B). On average there were 15 cellular objects per tile (range 0–43 objects). Prior to generation of the full annotated dataset, the time required to annotate each $1024 \times 1024$ tile was assessed for the four most popular annotation modalities: centre point (1 click per object), bounding box (2 clicks per object), 4-point extremity (4 clicks per object) and boundary segmentation ($>15$ clicks per object). Unsurprisingly, there was a direct correlation between the number of clicks required and average time required to annotate a tile (Fig. 1C). Centre point annotation was 77.1% faster than the next fastest modality, permitting an extra 22 tiles to be annotated per hour (centre point 51 vs bounding box 29 tiles per hour). Assessors also reported occasional trouble in estimating the edges of bounding boxes and thus needing extra time to adjust some annotations, which rarely occurred for centre point annotations.

**Figure 2:**
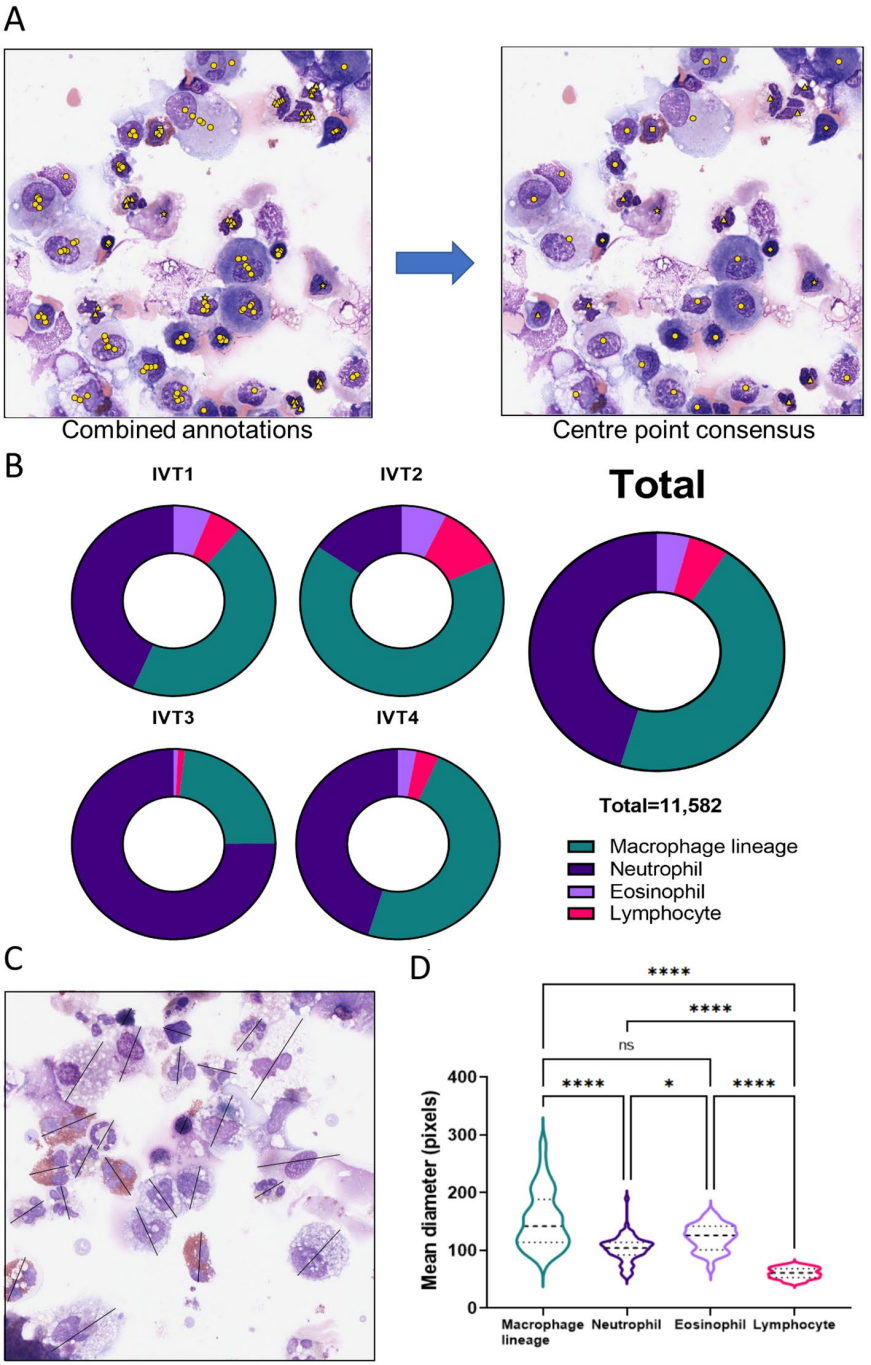
Final image label data used in the study. (**A**) Individual annotations by the four assessors were combined, determined by majority consensus. Symbols are in yellow for clarity, highlghting macrophage lineage (circle), neutrophil (triangle), eosinophil (square) and lymphocyte (diamond). (**B**) Final composition of the consensus annotated cell events. Data is presented for the four separate digital image datasets (each containing at least 160 images) used for interperson variability testing (IVT) and the total dataset of annotated 760 images. IVT1: 3 CF cytospins, 2 asthma; IVT2: 4 CF, 0 asthma; IVT3: 0 CF, 5 asthma; IVT4: 0 CF, 5 asthma. (**C**) Widths of cell objects were measured across the longest distance per object and recorded as number of pixels. (**D**) Immune cell classes were significantly different from each other (Kruskall–Wallis test with Dunn’s multiple comparison correction).

**Figure 3:**
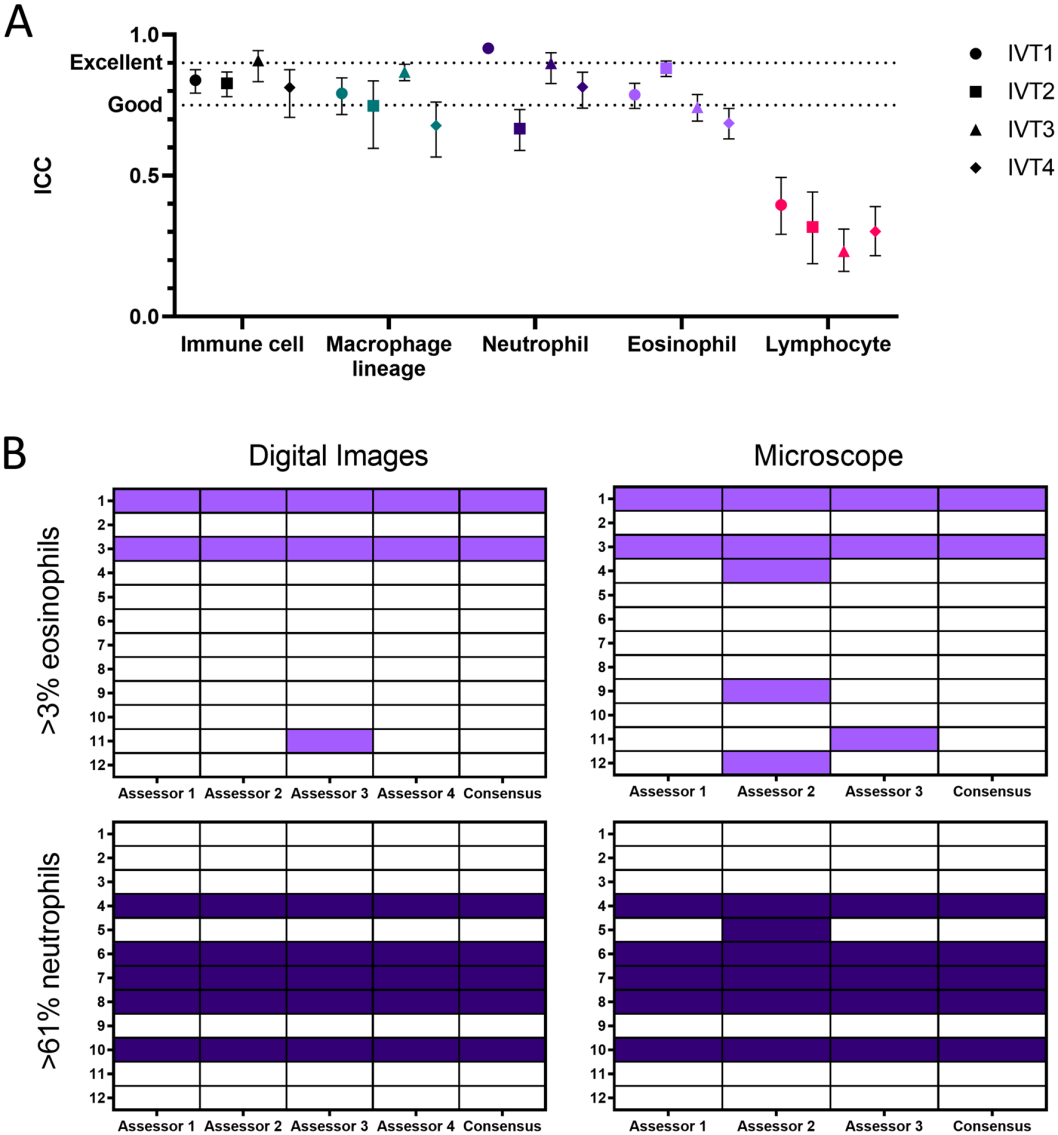
Assessor agreement at both object level and final cytospin determination. (**A**) Good agreement between the four assessors was achieved in all outcomes except identification of lymphocytes. Data represents four separate rounds of annotation and interperson variability testing (IVT), each performed using at least 160 digital images of cytospins. The agreement between the four assessors was calculated for each immune cell class (Macrophage lineage, Neutrophil, Eosinophil, Lymphocyte) as well as agreement at a total level (“Immune Cell”) and is presented as intraclass correlation coefficient (ICC). Lines indicate cutoffs for “good” (≥ 0.75) and “excellent” (≥ 0.90) agreement^22^. (**B**) Agreement in final assessor determination of asthma cytopsins according to criteria of either $>3\%$ eosinophils or $>61\%$ neutrophils (rows), based upon data from the digital image annotations or counts performed through physical viewing by microscope (columns). Closed cells indicate the source cytospin was determined to positively meet criteria threshold, open cells indicate negative determination.

### Establishing ground truth and evaluating human variability

Having established centre point annotations as the method most preferred by the team of four assessors (combined experience of 60 years), all 760 images were annotated for cell classification and centre points. Consensus between the four annotators was established (Fig. 2A) and resulted in 11,582 labelled immune cells (Fig. 2B). Macrophage lineage cells (45.4%) and neutrophils (45.35%) predominated over lymphocytes (5.07%) and eosinophils (4.2%). The composition of immune cell classes also varied greatly between the four rounds of annotation, as did the composition of the underlying clinical samples. The diameters of all objects in sample images were manually recorded as number of pixels across the furthest span (Fig. 2C) and distribution of cell diameters compared (Fig. 2D). The macrophage lineage population featured the largest median diameter as well as the largest range (median 142 pixels, interquartile range (IQR) 114–183 pixels, 102 objects measured), likely due to the known size differences between small monocytes and large alveolar macrophages. All classes were significantly different to each other (Kruskal–Wallis test with Dunn’s multiple comparison correction) with the exception of eosinophils, which were the second largest class (median 126 pixels, IQR 101–142 pixels, 31 objects measured) and not different to the macrophage lineage class ($\hbox {p}=0.27$). As expected, lymphocytes were the smallest cell class (median 61 pixels, IQR 53–68.5 pixels, 33 objects measured) and significantly smaller than neutrophils (median 104 pixels, IQR 92–114, 69 objects measured; $\hbox {p}<0.0001$).

Human variability is recognised in best-practice differential count assessments^1–3^. Intraclass correlation coefficient (ICC(A,1); absolute agreement ICC in the presence of bias) values for total immune cells and each class are shown in Fig. 3A, with ICC values presented separately for each round of IVT testing. There was consistently good assessor agreement ($\hbox {ICC} >0.75$^22^) for total immune cells (mean ICC $0.847 \pm 0.043$) across all IVT rounds. Overall assessor agreement was also good for macrophage lineage cells (mean ICC $0.772 \pm 0.080$), neutrophils (mean ICC $0.833 \pm 0.124$) and eosinophils (mean ICC $0.774 \pm 0.067$), though ICC for these classes could be highly variable between IVT rounds. The worst performing class was lymphocytes with a mean ICC of $0.312 \pm 0.067$, indicating poor agreement. A low ICC value of less than 0.5 was consistently observed for the lymphocyte class across all IVT rounds. Next, we investigated the agreement of assessors on how they classified asthma cytospins in terms of two categorical outcomes, $>3\%$ eosinophils and $>61\%$ neutrophils, as these are commonly reported in asthma and recently investigated for CellaVision${}^\circledR$ by Frøssing et al.^21^. Unique to this study, agreement was performed for both the digital annotation data generated from 40 image tiles per cytospin and a direct manual count of 400 cells through microscopy. Assessors demonstrated a strong consensus in categorising the 12 asthma cytopsins when based upon the image annotation data (Fig. 3B). Consensus was slightly lower when categories were allocated based upon manual count through a microscope.

### Differential count network (“DCNet”)

**Figure 4:**
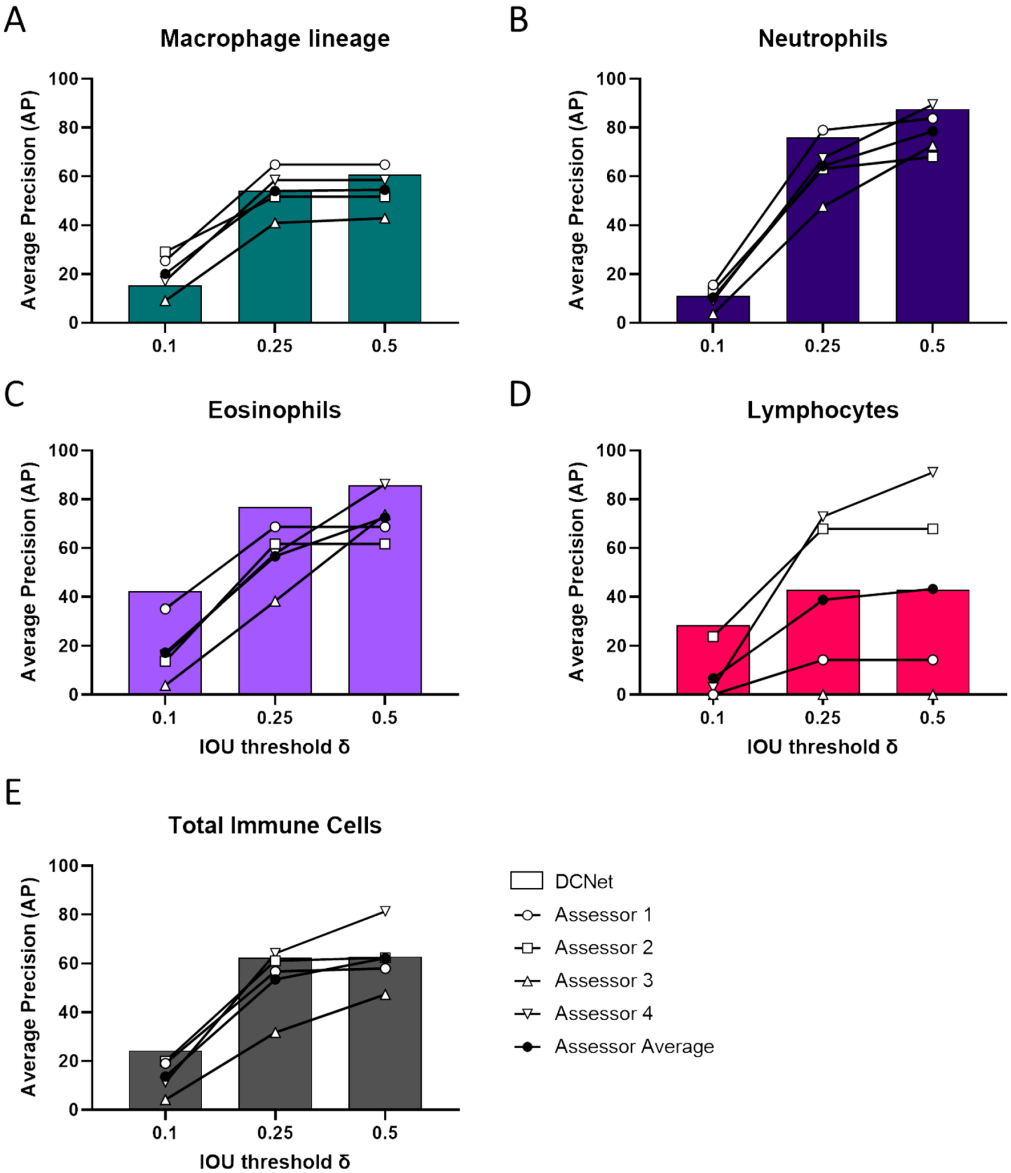
DCNet matches human assessors at immune cell classification. The average precision (AP) of DCNet predicted classifications was calculated as intersection over union (“IoU”) at thresholds to find true positives, relative to the distance from the ground truth in the cell centre. A $\delta = 0.5$ means the predicted centre point lies within half a cell $\sigma$ diameter away from the ground truth. This was also calculated for human assessors based upon their annotation and plotted for comparison for each immune cell class (**A–D**) and total immune cell population (**E**).

DCNet was trained using a fully convolutional encoder–decoder network to predict a centre point heatmap for each type of immune cell. Further details are provided in the Methods. To quantify the accuracy of the DCNet predictions, the average precision (“AP”) was computed by comparing the heatmap prediction against the consensus ground truth for the cell centre across a range of different maximal distance ($\delta$) thresholds from the cell centre (10%, 25%, 50% of cell size). The AP for both DCNet and human assessors decreased as the distance to ground truth threshold was reduced (Fig. 4). However, DCNet was able to achieve human baseline performance and matched or exceeded the average human AP, either at an individual object class (Fig. 4A–D) or total immune cell count (Fig. 4E). Despite macrophages and monocytes being the predominant cell class, both the DCNet and human AP for this class were lower than expected for the number of labelled objects, even at a $\delta$ threshold of 0.5. However, DCNet (AP 60.84%) still outperformed three of the four human assessors (average AP 54.5%). For both the neutrophil and eosinophil classes, DCNet displayed a consistently excellent AP across the $\delta$ thresholds (Fig. 4B,C). The most notable limitation was the lowest AP across all distance thresholds for the lymphocyte class (Fig. 4D). However, this was a consistent issue for the humans annotators (Fig. 3A) and DCNet at least matched the average AP of our human assessors. The low AP for DCNet in the lymphocyte class did not affect accuracy in identifying the total immune cell population, likely due to lymphocytes typically present at around 5%. In the most significant finding for the translational application of DCNet, we observed that DCNet outperformed the average human AP at all $\delta$ thresholds (Fig. 4E).

**Figure 5:**
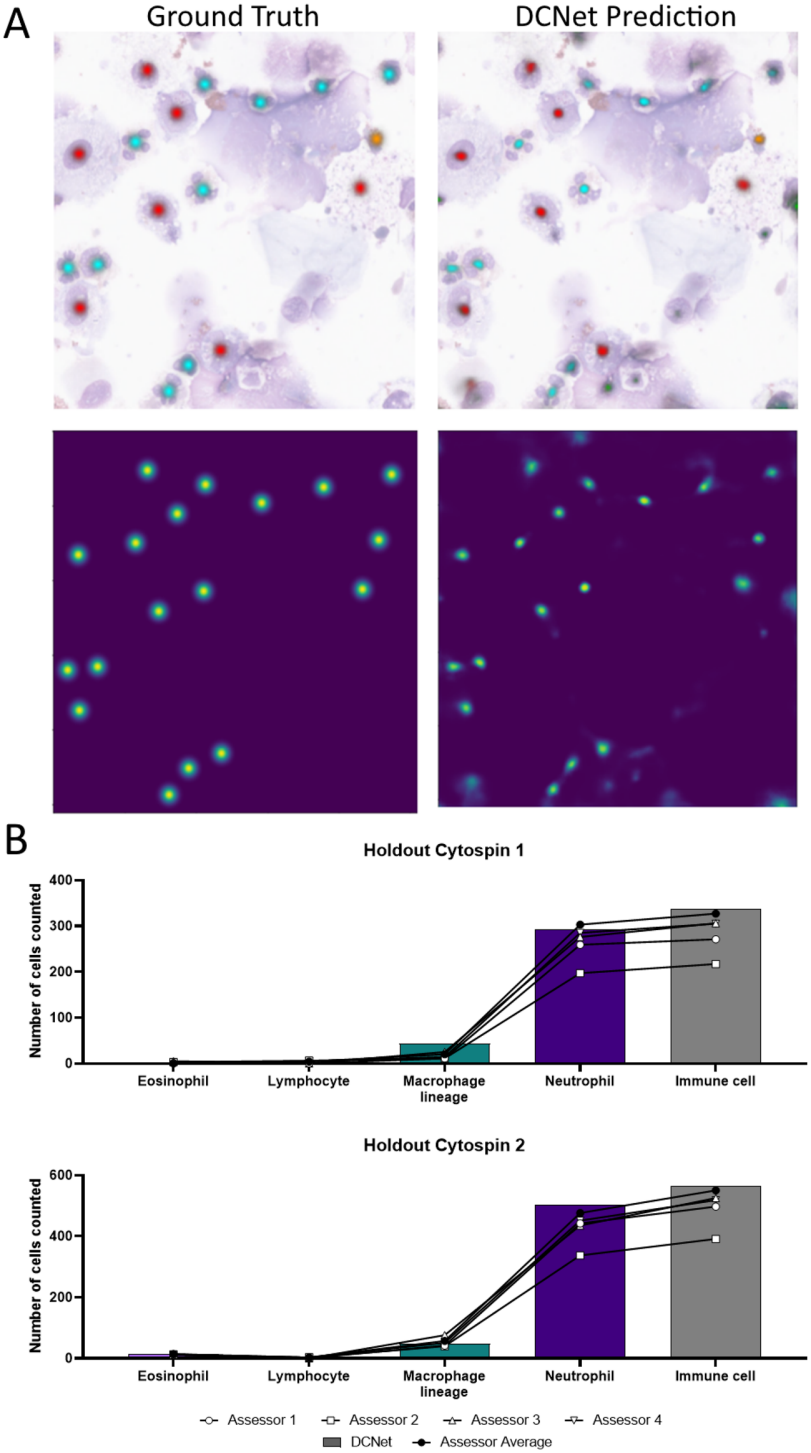
DCNet matches human assessors at total differential cell outcomes on unseen images. On two holdout cytospins not included in training, DCNet performed well in classification and localisation as indicated by (**A**) representative mask and heatmap images respectively. (**B**) DCNet also concurred with human annotation assessors on the final immune differential cell count for both holdout cytopsins. Data generated from 40 tile images per cytospin.

We then analysed the performance of DCNet on the holdout annotation dataset of 80 tile images from two cytospins, first by studying the DCNet heatmap predictions at an individual image level, comparing both the localisation and classification of predicted objects to the consensus ground truth data (Fig. 5A). Overall, the predictions were found to be well localised to ground truth with minimal false positives due to non-cellular debris such as mucous, which can contain extracellular DNA and present similar hues to nuclei following the histological stain. Given the model was trained with only 680 tiles (89.5% of total dataset), the predictions were encouraging and the performance was found to be consistent. Further examples are presented in Fig. S1. Most importantly, when the number of objects predicted in the 40 images per cytospin were summed, DCNet concurred with the human consensus counts (Fig. 5B) both in terms of individual class and total immune cells. Again, significant variation was observed in the human scores and particularly for eosinophils (range 0.33–1.63%).

To understand the immediate labour benefits that DCNet could provide in inflammation assessments, we assessed the rate at which DCNet could complete image analysis as an un-optimised, single thread computation with side-by-side image display. The average time for DCNet to predict cell classification counts for a block of 80 image tiles was 3.39 min, or 2.54 s per image tile. With our human assessors requiring an average of 70 second per tile (shown previously in Fig. 1C), DCNet performed 27.6× faster than expert assessors at immune cell classification. In our experience, a differential immune cell count on cytopsins viewed through a microscope are typically faster than the digital image annotation process and our human assessors could complete a 400 cell count in a mean time of 6.9 min, though maximal times can be as high as 9.4 min. In the current iteration as an unoptimised single thread operation, DCNet could classify 400 immune cells in approximately 1.7 min, a fourfold gain in efficiency with more consistent performance than human assessors.

### Basic cell detection on public benchmark data

Finally, we demonstrated DCNet on another multiple object detection problem by evaluating the performance on the pre-existing 2018 Kaggle Data Science Bowl (KDSB2018) dataset^23^. The KDSB2018 dataset contains 673 images of bright field and fluorescent cells with associated nuclei segmentation masks. It differs from our primary clinical sample dataset in that it features just a single object class and focuses on the nuclei only. However, the dataset includes robust segmentation labels which allowed us to benchmark against other segmentation and bounding box methods. The comparators selected were RetinaNet and Mask-RCNN. Retina-Net^24^ is a bounding-box detection network that uses a two-stage region proposal network. Mask-RCNN^25^ is an instance-segmentation based network used to predict both bounding boxes and segmentation masks. Both algorithms utilise an anchor-based two-stage network approach. The first stage being a region proposal network, the second stage being a classifier. In addition to testing DCNet on the KDSB2018, we also tested two further variants of DCNet with additional model architectures and loss functions to leverage the bounding box and segmentation annotations in KDSB2018.

For the RetinaNet and Mask-RCNN models, we calculated ${AP}_{\delta }$ by extracting the centre points of each respective predicted bounding box or segmentation mask. Table 2 shows the results of our evaluations on KSDB2018 across different $\delta$ thresholds. In terms of performance, DCNet outperformed RetinaNet across the board and compared favorably to Mask-RCNN, while offering considerable advantages in inference speed and reduced model complexity. Table 2 also shows that DCNet performed slightly better than two other key points networks variations, CenterNet-HG^18^ which uses Hourglass-104 architecture with slightly different modified focal loss functions parameters and DCNet-CE, in which used the same ResNet34 architecture as DCNet but with semantic segmentation method applied in place of heatmaps. The differences in predictions across the tested detection networks is shown in Fig. S2, illustrating the shortcomings of object detection networks when objects are clustered together, as is common in cytopathology images. In contrast, centre point detection was very well suited for this situation as while cell walls can often overlap or have unclear boundaries, cell centres never overlap and were rarely within close pixel proximity to each other.

**Table 2:** Benchmark results and model sizes in identifying cell nuclei in the 2018 Kaggle Data Science Bowl dataset.

Model name	AP ($\delta =0.1$)	AP ($\delta =0.25$)	AP ($\delta =0.5$)	F1 ($\delta =0.5$)	Trainable parameters
**DCNet**	57.6	**78.1**	**83.2**	0.861	20M
RetinaNet	12.9	60.5	82.9	0.648	36M
Mask-RCNN	**63.4**	75.0	79.0	**0.865**	44M
DCNet-CE	54.0	71.9	82.4	0.828	20M
CenterNet-HG	54.9	74.9	78.6	0.853	18M

The average precision (AP) of DCNet is compared against existing state of the art classifiers (RetinaNet^24^ and Mask-RCNN^25^) as well as two other key points networks variants of DCNet; DCNet semantic segmentation (DCNet-CE) and CenterNet hourglass network (CenterNet-HG). The AP for each model is reported across the percentage distance threshold values ($\delta$) of 10%, 25% and 50% cell width, along with F1 score at 50% cell width. Higher AP/F1 score is better and bold figures indicates the best performing model for each threshold. The number of parameters (*M* million) for each model is shown. Lower parameters is better with the number of parameters roughly proportional to time required for training the model.

## Discussion

Here we have demonstrated the development and capability of DCNet, a deep learning based image analysis algorithm that capitalises on highly efficient centre point ground truth labels to generate accurate multiclass object classification and counting. Our approach addresses both (1) the lack of efficient labelling modalities to assist field experts in maximising their output of annotated cell data and (2) variable performance of whole cell detection models that are sensitive to object density and the amount of non-object features. In this paper, we detailed an example pipeline of image acquisition of clinical cytopathology specimens and efficient annotation, highlighted how digital comparisons better quantify the inherent variability between expert human assessors and demonstrated how DCNet could be trained on relatively limited centre point annotation data to match human performance targets. Finally, in a controlled application at solving a more general cell identification problem on public benchmark data, we illustrated how DCNet exceeds existing bounding-box or segmentation based deep learning models.

Numerous algorithms have been developed for cell recognition. To date, the deep learning field working on cell orientated computer vision has largely taken a dichotomous approach, seeking to identify the cells in an image from the surrounding non-cell features. By focusing mainly on just the recognition of “any cell”, whose single defining feature uniform to all cells is the presence of a nucleus, the majority of cell image datasets that have been created are solely labelled with cell nuclei annotations and not any classification of cell sub-types^10,13,15,26,27^. The associated algorithms developed for these datasets are useful for quantifying a general cell count and associated metrics like cell density in an image, which can inform analysis of tumour histology images. Some prior approaches have classified cells into more than one class^9,28,29^, however these classifications were either centered around nuclei morphology, or required laborious annotation such as complete cell segmentation which has been demonstrated to work well for detecting cells^4,30^. Current state-of-the-art approaches such as Mask-RCNN^5,25^ use a combined approach of bounding box object detection and segmentation. Models utilising only bounding box annotation have also proven popular for cell recognition problems^5,16^. However, since cells on cytopathology images often vary significantly in the opacity of the internal structure and do not always feature high contrast boundaries, this can impact performance of bounding box networks as they are most efficient when cells are separated and distinct, with lower performance when cells are clustered together and/or surrounded with non-cellular structures such as debris or mucus^31^. From an annotation perspective, bounding boxes are more obviously attractive to pathology experts than segmentation techniques, requiring only 4-point annotations for the box. Yet because each box should be ideally sized to the smallest area possible within which all the points of the object lie, there is the potential for wasted labor when boxes need correction. Even semi-automatic annotation tools that are available^32–35^ can require more than 1-click and precise annotations on the edges of each cell object. Feedback from the annotation team highlighted that labelling centre points was less taxing over long labeling sessions and facilitated labeling tiles with high object density due to less visual clutter. Boundary segmentation was deemed too arduous by the annotation team. Even with rough segmentation rather than pixel level segmentation, no assessor was willing to complete segmentation labeling for the complete set of tiles.

We identified a particular need for automated differential immune cell counts in respiratory samples as this field has limited choices in terms of digital solutions^21^ that could facilitate improved monitoring of inflammatory respiratory conditions. Furthermore, there was a distinct lack of appropriate training data in the literature. Employing four experienced human assessors of respiratory differential immune cell counts, we have demonstrated that centre point annotation directly provides significant efficiency gains in ground truth data generation over the popular bounding box approach. We also used this opportunity to better quantify the existing human variation in immune cell counts. To our knowledge we are the first to measure assessor agreement on digital images of cytopathology samples rather than microscope attendance^2,3^. By comparing the annotation data of four assessors at an individual object level on the same set of digital images, which avoids the region selection issues that are known to influence outcomes^3^, we found that assessor agreement could vary greatly. This variation was dependent upon the composition of the source cytospins for the digital images (variation between IVT rounds) and the target immune cell class, with lymphocytes consistently featuring lower ICC than the other classes. Together these findings indicate that the use of total cell counts or class percentages by prior literature on respiratory cytopathology, which typically use at least twice as many (30–200) clinical samples^2,3,21^ as our study but only compare outcomes from microscope attendance, may not have accurately estimated human performance at differential cell count classification. Although these studies show good agreement on an overall basis, in this study we report how human variation can not only bias the object classification but also affect clinical determination. Critically, it provided DCNet with a true target baseline for human performance in classifying individual objects for each immune cell class.

For sub-classification on non-nuclei features, humans assessors take into factors like hue, saturation, structure and size when classifying cells on differential count stains. We validated that immune cell classes were significantly different to each other in mean pixel size. So, we built upon the existing keypoint detector for object detection^18^ to incorporate variable class radius as a component of DCNet. The overall result was that DCNet provided a simpler end-to-end system compared to current literature, enabling end-users to train a custom multi-class cell classification model with state-of-the-art performance. In short, DCNet outperformed benchmark models on the 2018 Kaggle Data Science Bowl dataset at nuclei identification. On our respiratory cytospin dataset, DCNet was able to achieve better AP for multiple cell classification (62.8) than those reported to date in the cell identification literature, such as the 31.6 for histological images and 57.0 for fluorescence images by Mask-RCNN and U-Net models^5^. Similar to human assessors, DCNet exhibited highest precision (85.7–87.7) on the neutrophil and eosinophil class. This may be because these two classes uniquely feature multi-lobed nuclei along with distinct pigmentation from the eosin stain that help better differentiate these two populations from the background. On key difference is that neutrophils tend to feature the lowest contrast cell boundary that could confound the network and this may explain why neutrophil AP was lower than for eosinophils at a $\delta$ threshold of 0.1, despite the neutrophil class having 10 times the number of labelled training data. The macrophage AP was lower than expected for a dominant object class (60.8). However, this class is highly challenging because standard practice is to count macrophages and monocytes as a single entity. We suggest their very broad morphological variation during the stages of activation, particularly in size and non-nuclei features such as cytoplasm uniformity, require more than the 5258 labelled examples and expect that further training data would increase the accuracy at predicting cell centres for this class. Similarly, the relatively poor AP for lymphocytes (42.9) likely reflects a combination of low lymphocyte number in the total count (587, 5.07% of total immune cells) and the lack of a distinct marker, such as eosin positive granules, which assists in the identification of the the equally rare eosinophil population. Labelling of cells with fluorophore-conjugated antibodies to inform classification could directly address these issues. However, to our knowledge there are no established protocols combining histological stains with fluorophore-conjugated antibodies. Further, the imaging platform we utilized does not feature fluorescent imaging capacity. A molecular labelling solution warrants exploration to facilitate increasing AP for macrophages and lymphocytes, particularly if AP is non-responsive to increased human-annotated training data.

Additional to the performance of DCNet, the centre point annotation also provides benefits by reducing the time burden for valuable experts to generate ground truth data on cell classifications. This is obviously attractive to the commercial sector by reducing development costs but benefits academic research as well, since contributions may often be during personal time. The low barrier of a single click in the centre of object also permits studies to use any multitude of image annotation platforms. Together the reduced labor involvement and platform independence mean studies could be easily designed to have multiple assessors generate the ground truth, apply dataset review through simple consolidation and quality control queries with minimal reviewer interaction, further improving dataset rigour. We are now exploring if labeling efficiency can be further increased by pre-labeling new differential cell count training images using DCNet. Furthermore, by utilizing DCNet in combination with the Polygon-RNN algorithm^32^, we’ve been able to extract segmentation masks from the predicted keypoints of DCNet. This automated approach towards segmentation labelling will provide valuable data for future studies. Overall, these promising results show that DCNet can match performance with state-of-the-art detection networks without the need for more arduous bounding box or segmentation mask annotation. Our objective is to make DCNet deployable as an end-to-end system that can be fully integrated into a workflow so institutions can realise major time-savings for pathology based studies and develop deep learning networks for other specialised medical computer vision problems.

## Methods

### Cytospin preparation and imaging

Cytospins were from archives of prior approved studies into respiratory disease. Briefly, bronchoalveolar lavage fluid was collected from children with cystic fibrosis (CF) as part of the the Australian Respiratory Early Surveillance Team for Cystic Fibrosis (AREST CF) program^36^, a study approved by the Child and Adolescent Health Service Ethics Committee. Induced sputum from adults with asthma was collected as part of clinical trials approved by the Hunter New England Human Research Ethics Committee. In both studies, cytopsins were prepared as part of their research protocol by centrifugating the cellular fraction of the airway specimen (approximately $4 \times 10^{4}$ cells) onto glass pathology slides. Cells were stained with Kwik-Diff™ (ThermoFisher Scientific, Australia) or May-Grumwald Giemsa stain according to standard practices. To generate maximal resolution images for the purposes of this study, cytospins were digitised at $\times$ 100 oil magnification using a ScanScope OS (Leica Biosystems, Australia) through the Centre for Microscopy, Characterisation & Analysis, The University of Western Australia. Images were saved in the standard SVS container file format. Each sample contained approximately ten thousand $1024 \times 1024$ pixel tiles and each file was up to 2.5 GB in size. We utilised 40 tiles ($1024 \times 1024$ pixel size and without overlapping boundaries) from each of the 19 digitised cytospins for use in the annotation assessment and training of the detection networks.

### Ground truth annotation

All assessors annotated the centre points of cells for the full 760 image dataset, using the LabelBox^37^ platform. To minimise assessor fatigue and assist with interperson variability testing (IVT), labelling was conducted in four rounds of annotation, each round containing at least 160 images that were sourced from four randomly selected cytopsins. In LabelBox, the $1024 \times 1024$ image tiles were presented in random order to assessors, who labelled any identifiable cellular objects as belonging to one of four immune cell classes; macrophage lineage, neutrophil, eosinophil and lymphocyte. We grouped macrophages and monocytes as a single macrophage lineage class, as this is often done in cytopathology practice due to their overlap in morphological features. Since digital image boundaries are fixed in comparison to microscope visualisation for manual counts, assessors were asked not to label cells if the nuclei was not sufficiently visible on the tile. Once completed, annotations from all four assessors were collected, overlaid and an automated query was performed to consolidate duplicated class annotations within a 10 pixel radius. For duplicated annotations in disagreement of classification, a majority wins approach was taken. Finally, our “ground truth” dataset was finalised by a single assessor (“Assessor 1”), who reviewed all 760 annotated images in the dataset to correct any further duplicate or conflicting annotations that were not addressed by the automated cleanup. This assessor also ensured all point annotations were located in *the centre of the complete cell object*, not the centre of the nuclei (Fig. 2A). This “expert-in-the-loop” annotation process avoided missing labels and offered consensus annotation when disagreement occurred. To understand the baseline human performance level that DCNet must achieve, agreement between the classifications by our individual assessors at the cellular object level was compared through ICC analysis.

### DCNet

The DCNet approach utilises a fully convolutional encoder–decoder network to predict a centre point heatmap for each type of immune cell. Our model is end-to-end differentiable and trained using a weighted pixel-wise logistic regression with focal loss. At inference time, we extract the centre clusters from each corresponding class mask and calculate average precision (AP) based on euclidean distance between predicted and ground truth centre points.

#### Center point adaptive radius heatmap

To overcome the lack of a defined object size in the annotation, which is a limitation of the centre point annotation method versus traditional bounding box or segmentation approaches, an approximation of the relative size differences between the classes was established. To generate our target heatmaps in Fig. 5, we create a set of masks $Y \in \ (0, 1) \, W\times H \times C$, where C corresponds to the number of cell types and W x H to the input resolution. A selection of 24 image tiles from across the 19 cytospin samples, based upon their lymphocyte or eosinophil counts, was analysed and the cell diameters measured in pixel units, as presented in Fig. 2C,D. Having determined the mean radius of each cell class, we plotted each ground truth cell point annotation using a 2D Gaussian kernel (1) onto the mask, placed at the cell centre and the variance $\sigma _C$ determined by the average radius for each cell class, C.

1 $$\begin{aligned} K_{\sigma _C}(x,y) = e^{-\frac{z^{2}+y^{2}}{2 \sigma _C^{2}}} \end{aligned}.$$

#### Architecture

**Figure 6:**
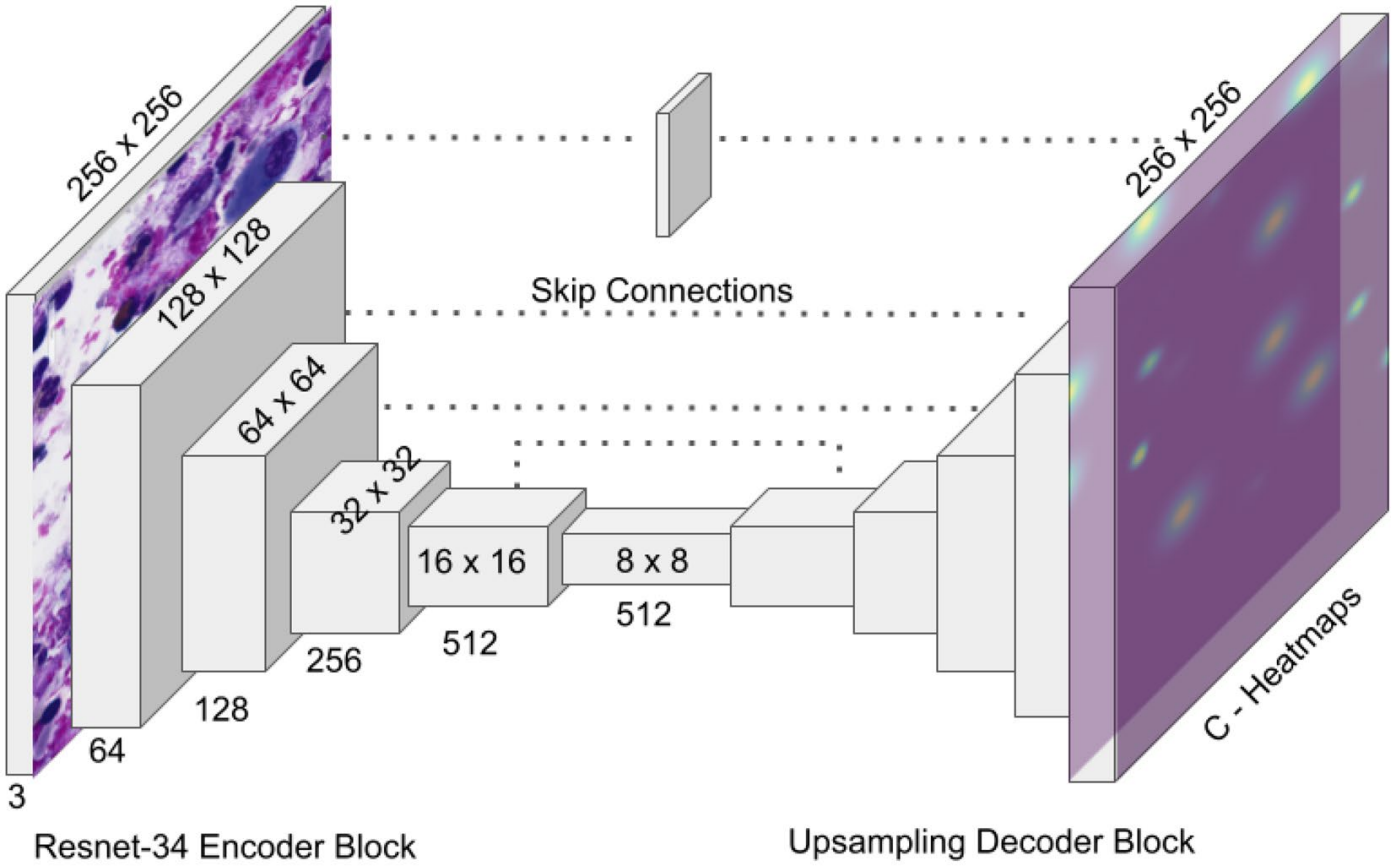
DCNet consists of a Resnet34 base encoder and 5 corresponding upsampling blocks. Input: $256 \times 256 \times 3$. Output: $256 \times 256 \times$ C heatmaps.

We used variations of the U-Net architecture to predict center point heatmaps. U-net, perhaps the most widely adopted by the bio-medical field since it was introduced in 2015^4^, consists of a fully-convolutional encoder-decoder network with skip connections. Our base model, which we refer to as ‘DCNet’, uses ResNet-34 as the encoder backbone (Fig. 6). The decoder consists of 5 upsampling blocks with a pixel shuffle upsampling layer and two $3\times 3$ convolutional layers, each followed by batch normalization. Specific model details are outlined in the fast.ai library^38^ which is developed at the Data Institute, University of San Francisco. Two variants of DCnet were also tested. CenterNet-HG uses an Hourglass-104 network most commonly used in keypoint detection networks^17,18^. This network consists of 2 downsampling layers, followed by 2 stacked hourglass networks. In addition, we adapted the base DCNet model to use a semantic segmentation approach, which we refer to as DCNet-CE. Instead of heatmaps, DCNet-CE uses a weighted categorical-cross-entropy loss to predict segmentation masks with object-centers. This approach utilised the same image preparation and loss function described in the publications by Falk et al.^6^ and Sirinukunwattana et al.^9^.

#### Loss function

We implemented a penalty-reduced logistic regression with focal loss as described by Zhou et al.^18^. A target ${Y}_{x y c} = 1$ corresponds to the heatmap centre point (x, y), while ${Y}_{x y c} = 0$ corresponds to the background, with a weighted negative loss based on the Gaussian distance (1) to each centre point. We set $\alpha$ with reference to the number of objects and $\beta$ to 2 in all of our experiments.

2 $$L = \frac{{ - 1}}{N}\sum\limits_{{xyc}} {\left\{ {\begin{array}{*{20}l} {\left( {1 - \hat{Y}_{{xyc}} } \right)^{\alpha } \log \left( {\hat{Y}_{{xyc}} } \right)} & {{\text{if }}Y_{{xyc}} = 1} \\ {\left( {1 - Y_{{xyc}} } \right)^{\beta } \left( {\hat{Y}_{{xyc}} } \right)^{\alpha } \log \left( {1 - \hat{Y}_{{xyc}} } \right)} & {{\text{otherwise}}} \\ \end{array} } \right.} ,$$

#### Training

Prior to training DCNet, two cytospins (10.5% of annotated images) were first set aside as the validation dataset. With the remaining images, we trained on a reduced input resolution of $256 \times 256$ to predict C heatmaps. Data augmentation includes random rotation, warp, color jittering, and horizontal/vertical flips. We used a fixed weight decay of $10^{-5}$ and a context specific optimal learning rate based on the use of learning rate finder^39^. Unless specified otherwise, hyper-parameters are consistent across all experiments and models trained for 60 epochs on an NVIDIA V100 GPU with 8G RAM.

Annotated image data and DCNet code is available at the following Github repository https://github.com/slee5777/DCNet.

#### Metrics

Conventionally, average precision (AP) is used to evaluate the performance of segmentation problem by calculating the intersection over union (“IoU”) across several thresholds to find true positives. The Dice coefficient is very similar to IoU and they are positively correlated^40^. Since the output predictions are (x,y) coordinates, we used Percentage of Correct Parts (PCP) to measure accuracy by using a modified method first introduced in pose estimation networks^41^. At inference time, we extracted the peaks for each predicted class heatmap and selected any clusters with a maximum cluster area of 16 and a prediction score greater than 0.5. For each cluster, we chose the point with the highest prediction as the object centre point.

Given a list of predicted object centres ${\hat{Y}}_{{x y}}^{(C)}$, for each class, we calculated true positives by comparing each point to the ground truth points ${Y}_{{x y}}^{(C)}$. A predicted point is counted as a true positive if it lies within a certain distance threshold $\delta$ of an unmatched ground truth centre point of the same class. Next, for each set of true positive predictions, we reported the AP for each class and compared them against our assessors.

The distance threshold was calculated by multiplying average size $\sigma$ of the cell class over a range of percentage threshold values $\delta \in [0.1, 0.25, 0.50]$. We evaluated our model across these thresholds $\delta$ and reported the AP. A $\delta = 1$ means the predicted centre point lies within one cell $\sigma$ diameter away from the ground truth. The AP was also calculated for the individual assessors by comparing their specific annotations to the final curated ground truth.

### Statistical analysis

Analyses were performed in R^42^ or GraphPad Prism version 9.0.1 for Windows, GraphPad Software, San Diego, California USA, www.graphpad.com. Data were subjected to normality testing by histogram plotting and D’Agostino-Pearson omnibus K2 test. Untransformed data were analysed using parametric or non-parametric analyses as indicated appropriately through the text. $\textit{p} < 0.05$ was considered statistically significant. Absolute agreement ICC in the presence of bias (named ICC(A,1)^43^) were performed with the ’irr’ package in R using the function ${<}{<}$icc(Ma, model=“twoway”, type=“agreement”)${>}{>}$.

## Supplementary Information

Supplementary Information.
