# Supplementary material for: Differential cell counts using center-point networks achieves human-level accuracy and efficiency over segmentation
Source: Sci Rep. 2021 Aug 19;11:16917. doi: 10.1038/s41598-021-96067-3 (PMC8377024; doi:10.1038/s41598-021-96067-3)
Supplement: Supplementary file 1 — Supplementary Information. [file 41598_2021_96067_MOESM1_ESM.pdf]

Supplementary Material

Differential cell counts using Center-Point networks achieves human-level accuracy and efficiency over segmentation

Sarada MW Lee[1,2,+], Andrew Shaw[3,+], Prof. Jodie L Simpson[2,4], Prof. David Uminsky[5,†], Dr. Luke W Garratt[6,*,†]

[1]Perth Machine Learning Group, Perth, WA 6000, Australia [2]School of Medicine and Public Health, University of Newcastle, Callaghan, NSW 2308, Australia [3]Data Institute, University of San Francisco, San Francisco, CA 94117, USA [4]Priority Research Centre for Healthy Lungs, University of Newcastle, Callaghan, NSW 2308, Australia [5]Department of Computer Science, University of Chicago, Chicago, IL 60637, USA [6]Wal-yan Respiratory Research Centre, Telethon Kids Institute, University of Western Australia, Nedlands, WA 6009, Australia

[+]these authors contributed equally as first authors
[†]these authors contributed equally as senior authors

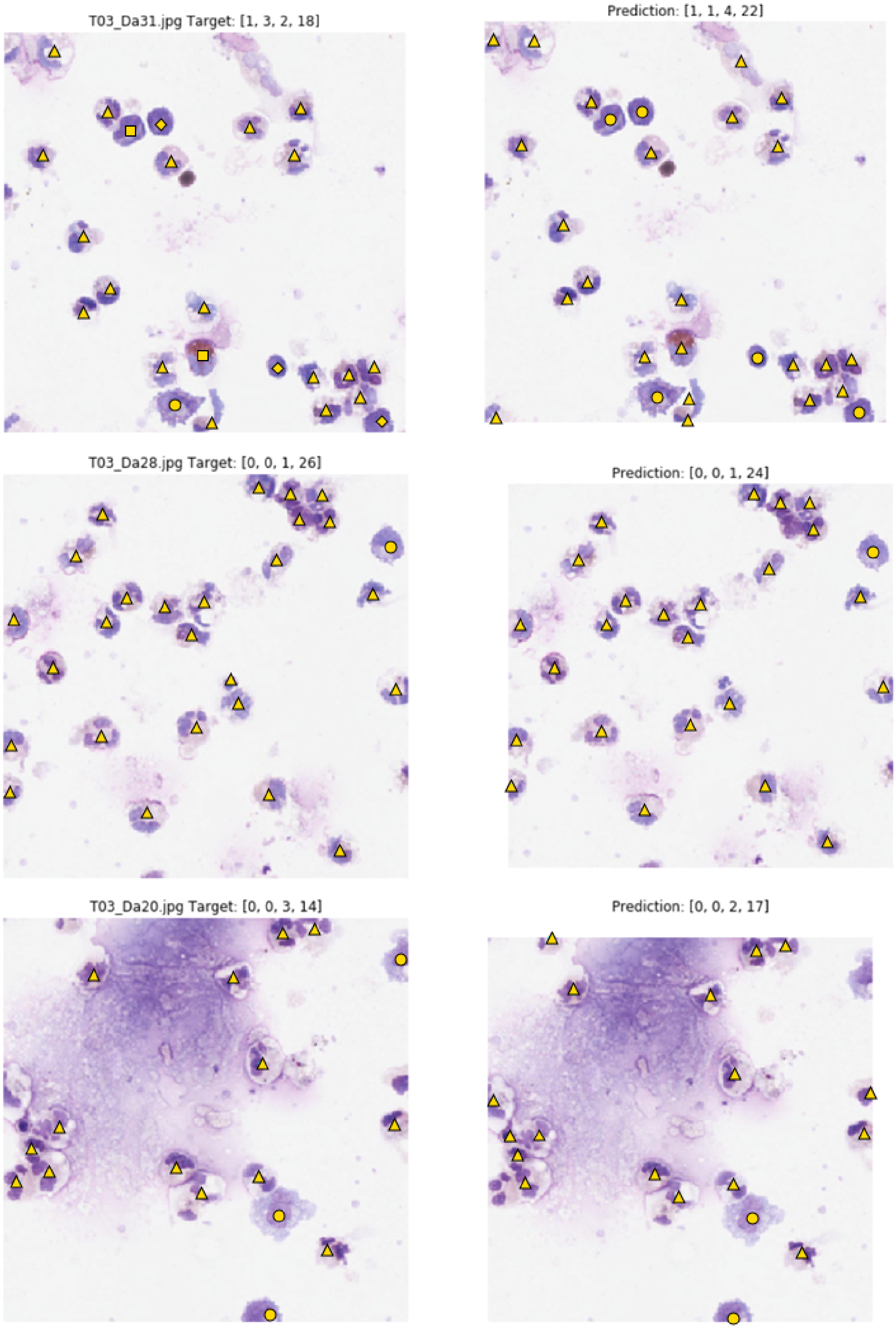

Figure S1. Representative images of DCNet performance on holdout cytospins. Left column: Target ground truth from assessor consensus annotation. Right column: cell location and classification predictions by DCNet. Symbols are in yellow for clarity, highlghting eosinophil (square), lymphocyte (diamond), macrophage lineage (circle) and neutrophil (triangle). Numbers in brackets reflect count of each class.

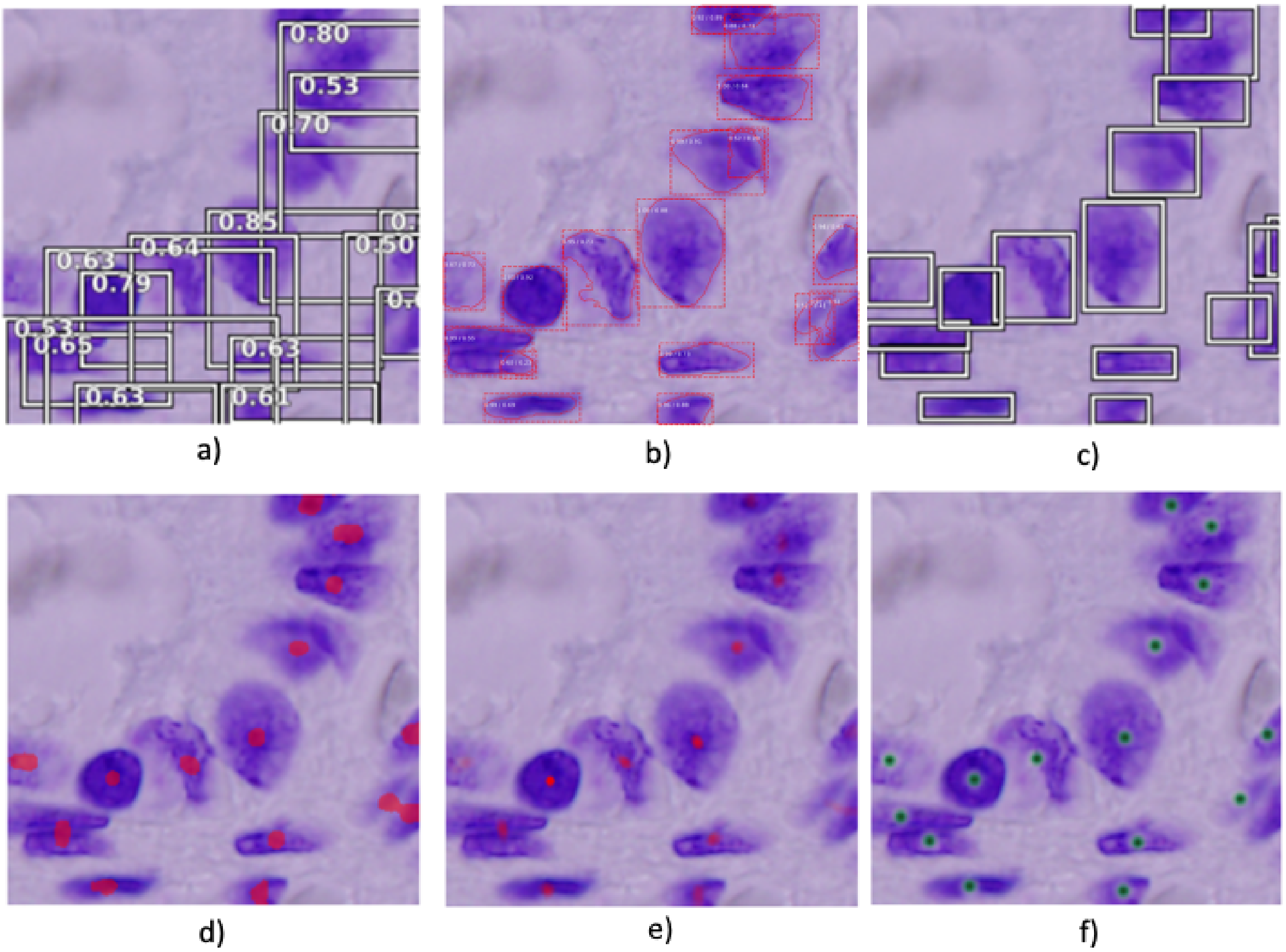

Figure S2. Comparison of detection networks for nuclei detection in the 2018 Kaggle Data Science Bowl dataset[23]. (**a**) RetinaNet predictions, (**b**) Mask-RCNN predictions, (**c**) bounding box ground truth, (**d**) DCNet-CE prediction, (**e**) DCNet predictions, (**f**) nuclei centre point ground truth.
